# Extracellular vesicles in tumor-adipose tissue crosstalk: key drivers and therapeutic targets in cancer cachexia

**DOI:** 10.20517/evcna.2024.36

**Published:** 2024-07-23

**Authors:** Cátia C. Ramos, José Pires, Esperanza Gonzalez, Clara Garcia-Vallicrosa, Celso A. Reis, Juan M. Falcon-Perez, Daniela Freitas

**Affiliations:** ^1^i3S - Institute for Research and Innovation in Health, University of Porto, Porto 4200, Portugal.; ^2^IPATIMUP - Institute of Molecular Pathology and Immunology of the University of Porto, Porto 4200, Portugal.; ^3^Institute of Biomedical Sciences Abel Salazar (ICBAS), University of Porto, Porto 4050, Portugal.; ^4^Faculty of Medicine, University of Porto (FMUP), Porto 4200, Portugal.; ^5^Exosomes Laboratory, CIC bioGUNE-BRTA, CIBERehd, Derio 48160, Spain.; ^6^IKERBASQUE Research Foundation, Bilbao 48009, Spain.

**Keywords:** Cancer cachexia, extracellular vesicles, cancer, adipose tissue transdifferentiation, metabolism, exosome

## Abstract

Cancer cachexia is a complex metabolic syndrome characterized by unintentional loss of skeletal muscle and body fat. This syndrome is frequently associated with different types of cancer and negatively affects the prognosis and outcome of these patients. It involves a dynamic interplay between tumor cells and adipose tissue, where tumor-derived extracellular vesicles (EVs) play a crucial role in mediating intercellular communication. Tumor cells release EVs containing bioactive molecules such as hormones (adrenomedullin, PTHrP), pro-inflammatory cytokines (IL-6), and miRNAs (miR-1304-3p, miR-204-5p, miR-155, miR-425-3p, miR-146b-5p, miR-92a-3p), which can trigger lipolysis and induce the browning of white adipocytes contributing to a cancer cachexia phenotype. On the other hand, adipocyte-derived EVs can reprogram the metabolism of tumor cells by transporting fatty acids and enzymes involved in fatty acid oxidation, resulting in tumor growth and progression. These vesicles also carry leptin and key miRNAs (miR-155-5p, miR-10a-3p, miR-30a-3p, miR-32a/b, miR-21), thereby supporting tumor cell proliferation, metastasis formation, and therapy resistance. Understanding the intricate network underlying EV-mediated communication between tumor cells and adipocytes can provide critical insights into the mechanisms driving cancer cachexia. This review consolidates current knowledge on the crosstalk between tumor cells and adipose tissue mediated by EVs and offers valuable insights for future research. It also addresses controversial topics in the field and possible therapeutic approaches to manage cancer cachexia and ultimately improve patient outcomes and quality of life.

## INTRODUCTION

Patients with cancer cachexia often experience unintentional weight loss mainly due to the progressive loss of body fat and skeletal muscle^[1,2]^. This metabolic disorder severely impacts patients’ quality of life, treatments, and survival^[3,4]^. Although nutritional supplementation is recommended in cancer cachexia patients, this solution is not capable of reversing cachexia symptoms^[5]^. Therefore, a better understanding of the mechanisms underlying cancer cachexia is essential to identify important players driving this syndrome to further improve patient’s clinical outcomes.

In cancer cachexia, the dysregulation between muscle protein synthesis and breakdown leads to substantial protein depletion within the skeletal muscle^[6,7]^. Recent research has highlighted the pivotal role of extracellular vesicles (EVs) derived from tumor cells in mediating communication between tumor cells, skeletal muscle, and adipose tissue (AT). By transporting key miRNAs, pro-inflammatory cytokines, and proteins, EVs can induce muscle wasting by modulating muscle physiology (as reviewed in refs^[8,9]^). This modulation includes myofibrillar protein degradation^[10-14]^, myoblast apoptosis^[15-17]^, insulin resistance^[18]^, and impaired mitochondrial function in muscle cells^[19,20]^.

As cancer progresses, tumor cells also establish a metabolic engagement with AT^[21,22]^. Tumor-derived EVs can induce the transdifferentiation of the white adipose tissue (WAT) into beige adipose tissue, resulting in global body weight loss^[21,23]^. As a feedback mechanism, mature adipocytes provide adipokines, lipids, and EVs [transporting proteins, fatty acids (FA), and lipid metabolism-related enzymes] to tumor cells, which consequently remodel their metabolism^[21,24]^.

Although skeletal muscle wasting is a major concern for cancer cachexia patients, this review focuses on the interaction between tumor cells and adipose tissue. We discuss and highlight the recent developments involving the role of EVs in mediating the tumor-adipose tissue (tumor-AT) crosstalk in a cancer-associated cachexia context. Furthermore, we provide valuable insights into the potential of EVs as emerging therapeutic targets, highlighting strategies to inhibit either the release or uptake of these nanovesicles and how this could delay the progression of cancer cachexia. In addition, we also provide promising findings regarding the use of engineered EVs to induce tumor cell apoptosis, reduce tumor progression, and enhance the response to therapy.

## CANCER CACHEXIA

More than half of all advanced cancer patients will experience cachexia at any point in their disease^[1-4]^. Moreover, cachexia is already considered to be the primary cause of death in 20%-30% of all cancer patients^[5,25,26]^. The incidence of this metabolic syndrome is most prevalent in gastric, pancreatic, lung, oesophageal, hepatic, and colorectal cancer patients^[3,27,28]^. Cachectic patients may experience asthenia, fatigue, anorexia, intestinal malabsorption, nausea, anemia, and a severe disorder in the metabolism of proteins, lipids, and carbohydrates^[29,30]^. In addition, these patients become more vulnerable to the toxic effects of treatments^[31]^.

Despite the severity of this condition, cancer cachexia remains an underdiagnosed condition due to the lack of standardized diagnostic criteria or biomarkers^[32]^. Indeed, biomarkers can provide important insights into the metabolic changes associated with cachexia, enabling early detection and personalized interventions^[5,33,34]^. Regular clinical follow-up and monitoring of cachexia parameters are essential for early detection, intervention, and management of this syndrome to optimize patient outcomes and quality of life. Despite the potential of biomarkers, there are currently no definitive biomarkers available for the diagnosis and management of cancer cachexia (as reviewed in ref^[33]^). Clinical monitoring of cachexia requires regular assessments of several parameters, including body weight loss and composition, inflammation [such as increased interleukin-6 (IL-6) or C-reactive protein], metabolic disturbances (such as anemia or low serum albumin), immunosuppression (such as low absolute lymphocyte number), decreased muscle strength and physical performance, fatigue, and anorexia^[5,32,35]^. Upon diagnosis, interventions may include nutritional supplementation, exercise programs, pharmacological therapies, symptom management, and multidisciplinary care^[5,36-38]^. Currently, the available recommendations for the management of cancer cachexia include a variety of drugs and hormones to (1) improve appetite and reduce nausea (including megestrol acetate, corticosteroids, and ghrelin hormone) (as reviewed in ref^[5]^); (2) increase lean body mass (including selective androgen receptor modulators and omega-3 FA); and (3) decrease inflammation (such as non-steroidal anti-inflammatory drugs)^[29,30]^. Unfortunately, the benefits and effectiveness of these strategies are limited and insufficient. Recent research has been focused on GDF15, a circulating growth factor associated with several types of cancer including prostate, gastric, colon, pancreas, and breast^[39,40]^. It is reported that high levels of GDF15 could promote tumor cell proliferation and metastasis^[40]^. Additionally, some studies have shown that high levels of GDF15 are associated with weight loss in animal models^[41,42]^ and poor survival rates for cancer patients^[43]^. Interestingly, treating mice with monoclonal antibodies targeting GDF15 could reverse the tumor-induced metabolic changes and promote weight gain in these animals, even with caloric restrictions^[44,45]^. Currently, several clinical trials are ongoing to evaluate the therapeutic potential of using GDF15 as a target for cancer cachexia. So far, these treatments have been well-tolerated by cancer patients and have resulted in a reduction in the circulating levels of GDF15^[46,47]^. These findings suggest that targeting the GDF15 growth factor may represent a promising therapeutic strategy for treating cancer cachexia.

In cancer cachexia, the skeletal muscle undergoes significant metabolic and inflammatory changes (as reviewed in refs^[38,48]^). Tumor cells release pro-inflammatory cytokines, such as tumor necrosis factor-α (TNF-α), IL-1β, and IL-6, which promote muscle protein degradation and increase oxidative stress^[49-51]^. Moreover, during this crosstalk, there is also evidence of (mTOR) signaling pathway inhibition^[52]^, insulin and insulin-like growth factor-1 (IGF-1) resistance^[53,54]^, and mitochondria dysfunction^[55]^. These combined effects result in muscle weakness, fatigue, and reduced response to cancer treatments, which severely impacts patient’s quality of life. Apart from the impact on skeletal muscle, cachexia can also affect the AT, heart, liver, brain, and immune system (as reviewed in refs^[1,56]^). Over the past decade, the communication between tumor cells and adipocytes has attracted researchers’ attention as it might provide new targets for addressing cancer cachexia. In particular, the changes occurring in AT morphology and function during cancer progression are associated with the development and progression of this metabolic syndrome.

## ADIPOSE TISSUE

**Figure 1:**
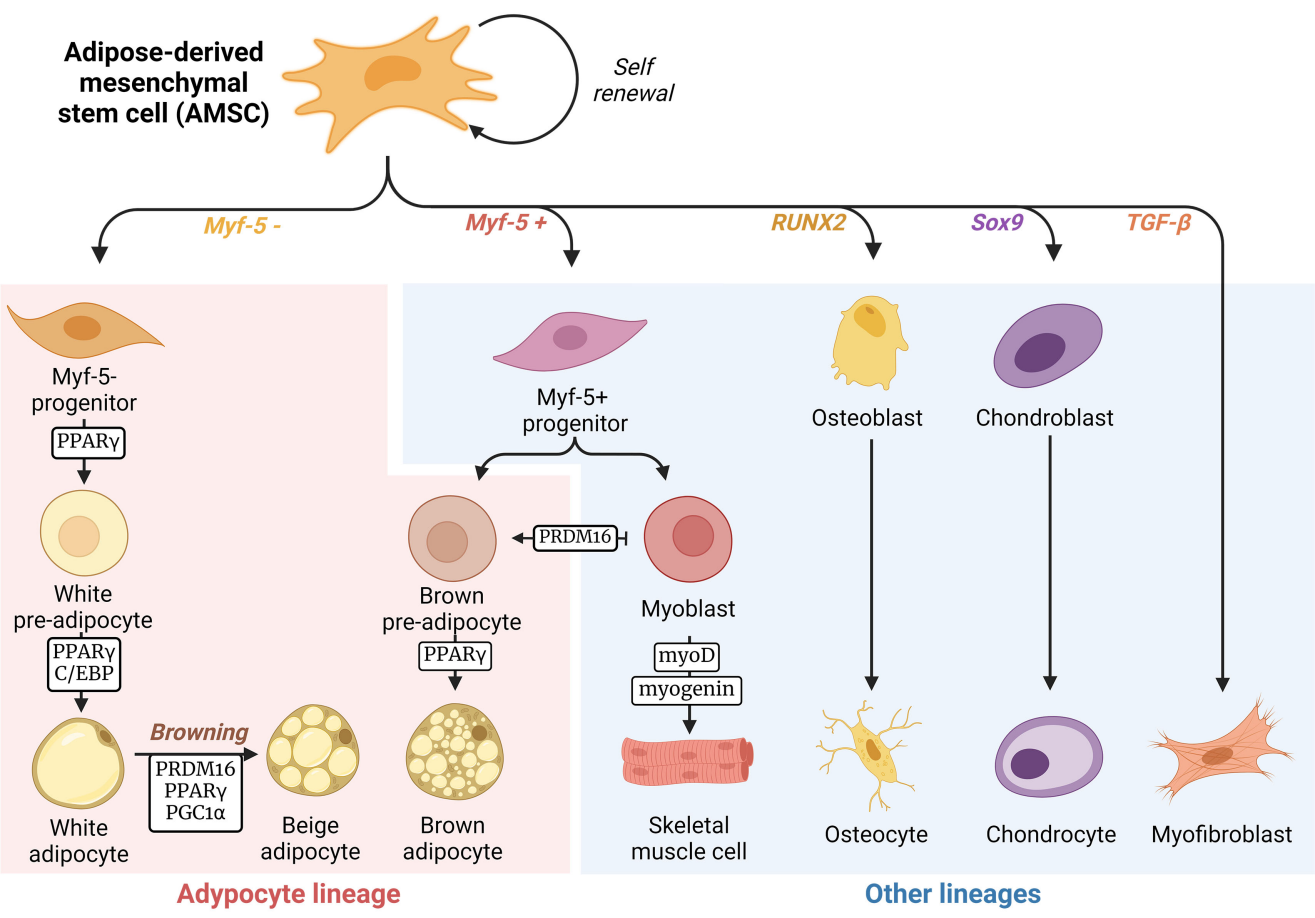
The differentiation process of adipocytes.AMSCs are mesenchymal stem cells that can differentiate into a variety of lineages depending on specific transcription factors. When the Myf-5 factor is absent and the PPARγ and C/EBP proteins are present, AMSCs differentiate into white pre-adipocytes. Upon fat uptake and deposit formation, these adipocytes further differentiate into mature white adipocytes. White adipocytes can also transdifferentiate into beige adipocytes, in a process called white adipocytes browning, in the presence of PRDM16, PPARγ, and PGC-1α. On the other hand, the presence of Myf-5 leads AMSCs to commit to a myogenic lineage. In this lineage, the PRDM16 transcription factor potentiates the AMSC differentiation into brown pre-adipocytes and prevents AMSC differentiation into myoblasts, and further skeletal muscle cells (due to myoD protein and myogenin). In the presence of PPARγ, brown pre-adipocytes mature into brown adipocytes. Other transcription factors, like RUNX2 and Sox9 factors, lead to osteogenic and chondrogenic lineages, respectively. There are also reports that TGF-β released by tumor cells also allows AMSCs to differentiate into myofibroblasts. Created with Biorender.com. AMSCs: Adipose-derived mesenchymal stem cells; Myf-5: myogenic factor 5; PPARγ: peroxisome proliferator-activated receptor-gamma; C/EBP: CCAAT/enhancer binding protein; PRDM16: PR domain containing 16; PGC-1α: PPARgamma-coactivator-1; RUNX2: runt-related transcription factor 2; Sox9: SRY-Box Transcription Factor 9; TGF-β: transforming growth factor-beta.

AT is a complex connective tissue composed of adipocytes and a stromal vascular fraction, which includes various cell types such as fibroblasts, pre-adipocytes, endothelial cells, and immune cells (as reviewed in refs^[57,58]^). In healthy individuals, the AT comprises around 25% of the total body weight^[59]^. Adipocytes are lipid-rich cells that contain globules of fat (lipid droplets) surrounded by a structural network of fibers^[59,60]^. Based on their morphological features, location, and function, adipocytes can be classified as white, brown, and beige adipocytes. The progenitors of white, beige, and brown adipocytes are mesenchymal stem cells derived from the AT, commonly known as adipose-derived mesenchymal stem cells (AMSCs). The importance of these AMSCs has only recently been studied, as many of their mechanisms remain unknown. In physiological conditions, this cell population can give rise to many different lineages^[61]^, including adipocytes, osteoblasts, or chondroblasts [Figure 1]. The differentiation process occurs in a synchronized manner through the regulation of a set of lineage-specific transcription factors. These include the peroxisome proliferator-activated receptor-gamma (PPARγ) for adipogenic lineage^[62]^, runt-related transcription factor 2 (RUNX2) for osteogenic lineage^[63]^, and SRY-Box Transcription Factor 9 (Sox9) for chondrogenic lineage^[64]^. Adipocyte cell lineage begins with AMSC differentiation. AMSCs can either be positive or negative for the myogenic factor 5 (Myf5). Myf5- AMSCs differentiate in the presence of PPARγ and CCAAT/enhancer binding protein (C/EBP) in white pre-adipocytes, which, upon cell cycle exit, can accumulate and form fat deposits as mature adipocytes^[65]^. Moreover, white adipocytes can also transdifferentiate into beige adipocytes in the presence of the PR domain containing 16 (PRDM16), PPARγ, and PPARgamma-coactivator-1 (PGC-1α). On the other hand, Myf5+ AMSCs give rise to precursors of the myogenic lineage^[66]^, which, upon the available transcription factors, differentiate in either brown pre-adipocytes, in the presence of PPARy^[65]^, or skeletal muscle cells, in the presence of myoblast determination protein 1 (myoD) and myogenin^[67]^.

**Figure 2:**
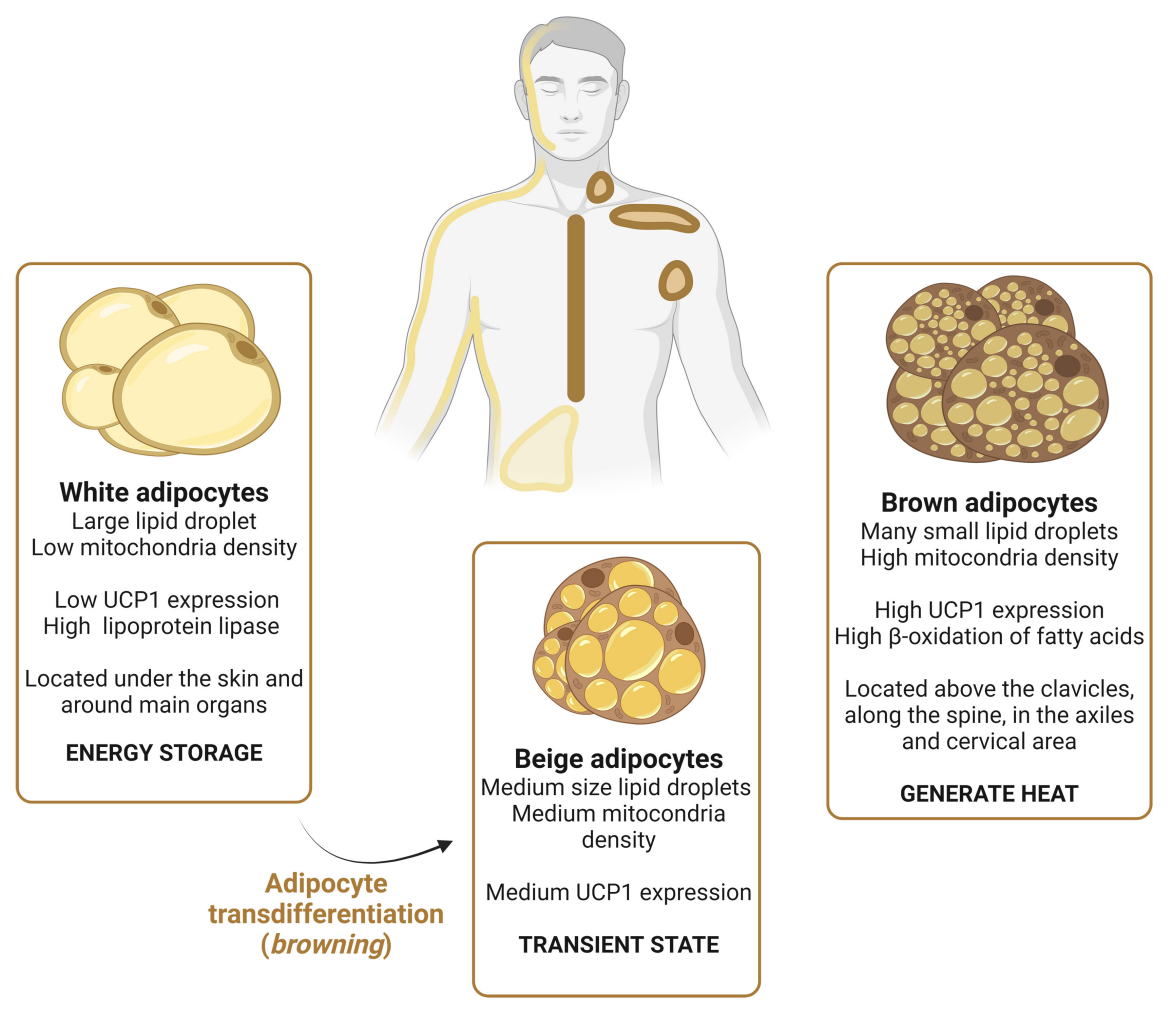
The three types of adipocytes. White adipocytes are characterized by a large lipid droplet and low mitochondria density and UCP1 expression. These adipocytes are responsible for energy storage, being capable of incorporating FA. They are mainly located under the skin and around the visceral organs. Due to a variety of stimuli, white adipocytes can transdifferentiate into beige adipocytes. Beige adipocytes have medium size lipid droplets and mitochondria density. Brown adipocytes are very metabolically active, being characterized by a high β-oxidation activity, sustained by a high mitochondria density. Due to this metabolic signature, they contain several small lipid droplets and very high UCP1 levels. These adipocytes are responsible for generating heat. Brown adipocytes are located above the clavicles, along the spine, in the armpits, and cervical area. Created with Biorender.com. UCP1: Uncoupling protein 1; FA: fatty acids.

Figure 2 represents the three types of adipocytes, their location, and their main function. White adipocytes comprise the majority of the body fat and function mainly to store energy. They are composed of a large lipid droplet and contain very few cellular organelles^[59]^. Therefore, their metabolic activity is very low. During lipogenesis, glucose is transported to the white adipocytes to be catabolized and transformed into FA^[1,68]^. FA are esterified with glycerol, giving rise to triglycerides, which are then stored in the lipid droplets of white adipocytes. These adipocytes are also capable of incorporating FA through the activity of lipoprotein lipase (LPL), the enzyme responsible for the degradation of triglycerides present in circulating lipoproteins. These FA can also be further esterified to triglycerides^[1,68]^. White adipocytes secrete key factors (hormones, growth factors, and cytokines) that play an important role in endocrine and metabolic regulation^[69]^. These adipocytes are located under the skin and around internal organs. On the other hand, brown adipocytes are very metabolically active cells containing many small lipid droplets, due to the β-oxidation of FA. Therefore, they are characterized by high mitochondria density, and positivity to the uncoupling protein 1 (UCP1)^[70,71]^. Their main function is to generate heat^[70]^ and are located in the upper back, above the clavicles, and along the spine^[72]^. During cold exposure, brown adipocytes are activated and white adipocytes can be transdifferentiated to acquire a brown fat-like phenotype, a process commonly known as white adipose tissue browning (WAT browning), giving rise to beige adipocytes^[73]^. As a consequence, an increase in the lipolysis activity^[74,75]^, and thermogenesis^[76,77]^ of white adipocytes, together with impaired adipogenesis and lipogenesis^[78,79]^, takes place. During WAT transdifferentiation, individuals may experience profound white adipocyte atrophy characterized by a series of morphological and structural modifications (as reviewed in ref^[80]^).

## THE TUMOR-ADIPOSE TISSUE CROSSTALK

Tumor cells can engage and induce WAT transdifferentiation into beige AT to stimulate lipolysis in adipocytes, in order to sustain their accelerated energy expenditure. Increased activity of adipocyte triglyceride lipase (ATGL), hormone-sensitive lipase (HSL), and monoglyceride lipase (MGL) enzymes^[81,82]^ results in the activation of lipolysis in white adipocytes. These enzymes are responsible for the hydrolysis of triglycerides. The resulting FA and glycerol are further released into the bloodstream and can be uptake by tumor cells^[83]^. As a result, tumor cells can adapt their metabolism toward FA β-oxidation (FAO) for energy production^[84]^. In addition, the high levels of ATGL and MGL have also been correlated with increased tumor aggressiveness in different types of cancer^[85-88]^. Furthermore, when tumor cells induce the WAT transdifferentiation into beige adipocytes, an increase in UCP1 levels can be observed^[89]^. By disrupting the mitochondrial ATP synthesis favoring thermogenesis, UCP1 leads to inefficient energy expenditure. This process culminates in involuntary body fat and weight loss, commonly found in cancer cachexia syndrome^[89]^. The synthesis of UCP1 protein is highly regulated by the PRDM16 protein^[90]^, PPARγ and PPARα proteins^[91]^, C/EBP-α^[91]^, CREB-binding protein^[92]^, and PGC-1α^[93,94]^. Subcutaneous cell implantation of cells displaying the lipid mobilizing factor zinc-α_2_-glycoprotein (ZAG) in mouse models stimulated the lipolysis and transdifferentiation of WAT^[95]^. The authors reported that the ZAG factor, highly expressed in several types of cancers^[96-98]^, could induce the activation of PPARγ and early B cell factor 2 (EBF2) and the recruitment of these factors to the PRDM16 promoter and consequently increase the levels of UCP1 protein^[95]^. Moreover, it was observed that the weight loss of colon-26 tumor-bearing mice, by adipose tissue wasting, begins in the early stages of cancer cachexia^[75]^. Notably, a positive correlation was found between the levels of UCP1 protein and basal lipolysis rate in the interscapular brown AT (BAT) of these mice^[75]^. In addition, the intravenous injection of mice with a lipid-mobilizing factor isolated from the urine of cachectic adenocarcinoma patients led to a marked decrease in their weight, together with a decrease in the plasma leptin levels and an increased UCP1, UCP2, and UCP3 levels in BAT^[99]^.

**Figure 3:**
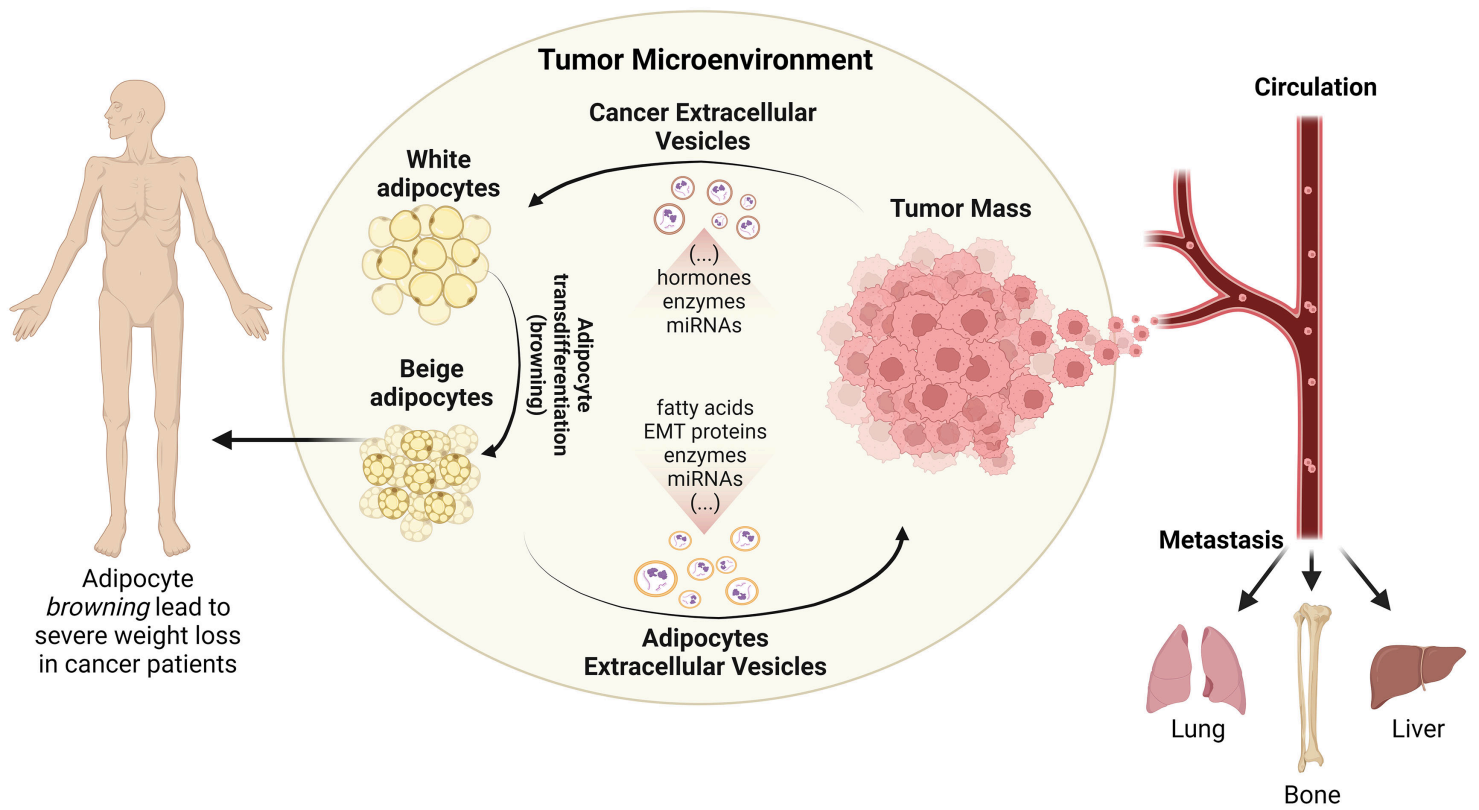
The tumor-adipose tissue crosstalk. Tumor cells are capable of remodeling adipocyte metabolism, locally and at a distance. Cancer cells secrete EVs containing a vast content of proteins and miRNAs that can promote the transdifferentiation of white adipocytes into beige adipocytes. In turn, the transdifferentiated white adipocytes fuel tumor growth and metastasis formation by engaging in a metabolic crosstalk with tumor cells. These adipocytes can secrete EVs enriched in specific metabolites, epithelial and mesenchymal markers, and/or miRNAs. As a consequence, tumor-AT crosstalk favors cancer progression and metastization of tumor cells to distant organs and induces cachexia in cancer patients. Created with Biorender.com. EVs: Extracellular vesicles; tumor-AT: tumor-adipose tissue.

Tumor cells can secrete numerous pro-inflammatory factors such as TNF-α, interferon-gamma (IFN-γ), IL-1 and IL-6, and parathyroid hormone-related protein (PTHrP) that can trigger cancer cachexia^[77,100,101]^. Notably, a positive correlation between the serum levels of IL-6 and free fatty acids (FFA) was found in gastric and colorectal cancer patients with cachexia^[102]^. Moreover, an increase in WAT lipolysis and browning in both early- and late-stage cachexia was observed in these patients. In addition, elevated levels of UCP1 and PGC1α were detected in the subcutaneous AT of gastrointestinal cancer patients^[103]^. These findings highlight the complex interplay between cancer-related inflammation and metabolic reprogramming in AT, contributing to the development and progression of cancer cachexia. Importantly, it is also known that tumor cells release EVs that can regulate tumor progression, metastasis formation, and chemoresistance by promoting intercellular communication (as reviewed in ref^[104]^). Recently, the role of these nanovesicles in mediating cancer cachexia has also been explored [Figure 3].

Recent research has provided important insights into the contribution of EVs to the development of cancer cachexia and tumor progression (as reviewed in refs^[9,105]^). Tumor-derived EVs carry several bioactive molecules like hormones, proteins, and miRNAs, which may induce lipolysis in AT, contributing to cancer cachexia^[106-109]^. Moreover, EVs can promote WAT browning by delivering pro-inflammatory cytokines and other signaling molecules to adipocytes, leading to increased expression of browning markers and enhanced thermogenesis^[11,110]^. On the other hand, EVs derived from adipocytes carry fatty acids, proteins, enzymes, and miRNAs that influence the behavior of recipient cells (as reviewed in ref^[111]^). These EVs can promote an inflammatory microenvironment favorable to tumor growth, invasion, and metastasis formation^[112-115]^. In addition, adipocyte EVs deliver lipids and lipid metabolic enzymes, thereby altering the metabolic state of tumor cells and promoting their proliferation and resistance to apoptosis^[116,117]^. Furthermore, adipocyte-derived EVs carry miRNAs capable of regulating gene expression in recipient tumor cells, often favoring tumor progression by inducing cell proliferation, invasion, and resistance to chemotherapy^[118-120]^. In summary, the role of EVs in tumor-AT crosstalk highlights their pivotal function in fostering tumor progression and ultimately leading to a cancer cachexia phenotype. Although further studies on different cancer types are still missing to strongly support the role of EVs in driving cancer cachexia, the existing findings already emphasize the potential use of EV-targeted therapies to tackle cancer cachexia.

### Cancer extracellular vesicles promote white adipocyte transdifferentiation and metabolism remodeling

EVs are a highly heterogeneous group of nano-sized particles that are essentially secreted by all types of cells as an intercellular communication mechanism. They travel in different body fluids, such as blood, saliva, tears, and breast milk, transporting proteins, enzymes, nucleic acids, lipids, and glycans to recipient cells (as reviewed in refs^[121-123]^). These vesicles can be classified by size as either small or large EVs, or by their biogenesis mechanism as exosomes, microvesicles, and apoptotic bodies^[124]^. Exosomes are produced by the inward budding of the cell membrane, whereas microvesicles are shed directly from the cell membrane^[125,126]^. Regarding apoptotic bodies, they are released during the process of cell apoptosis^[125,126]^. More recently, a subset of smaller nanoparticles called exomeres have also been described as secreted by cells with a differential cargo and function compared to small and large EVs^[127]^. EVs membrane consists of a phospholipid bilayer that confers protection to their cargo against proteases and nucleases present in the outside environment^[128,129]^. The small size of EVs^[127,130]^ facilitates their interaction and uptake by local cells and by cells in distant organs, where they can influence the behavior of the recipient cells^[104]^.

As previously mentioned, cancer cachexia is a multifactorial syndrome driven by several tumor-derived factors, including soluble cytokines and EVs. Tumor cells can release large amounts of pro-inflammatory cytokines, including TNF-α, IL-6, and IL-1β, into the bloodstream^[131]^. These cytokines induce systemic inflammation and catabolic processes in multiple tissues, leading to muscle wasting and fat loss^[131,132]^. In addition to cytokines, EVs have emerged as key players during cancer cachexia. Namely, it has been demonstrated the presence of miRNAs, hormones, proteins, and cytokines in cancer EVs that could induce a metabolic remodeling of white adipocytes after internalization. These vesicles can stimulate WAT lipolysis and browning, inhibit adipogenesis, and consequently lead to weight loss in animal models, as detailed in Table 1.

**Table 1:** Effects of cancer extracellular vesicles in white adipocyte transdifferentiation and metabolism remodeling

**Cancer type**	**Cancer EV cargo**	**Biological impact**	**Ref.**
Breast	miR-1304-3p	↑ Adipocyte “browning”	[133]
Breast	miR-204-5p	↑ Adipocyte lipolysis ↓ Mouse weight	[106]
Breast	miR-155	↓ Adipocyte lipogenesis ↓ Mouse weight	[134]
Pancreatic	Linc-ROR	↑ Dedifferentiation of white adipocytes	[135]
Pancreatic	Adrenomedullin	↑ Adipocyte lipolysis	[136]
Pancreatic	-	↓ Triglyceride content ↑ Glycerol release ↓ Mouse abdominal adipocyte size	[137]
Gastric	miR-410-3p	↓ Adipocyte adipogenesis	[138]
Gastric	miR-155	↓ AMSC adipogenesis ↑ Adipocyte “browning” ↓ Mouse weight	[110]
Gastric/Lung	miR-425-3p	↓ Pre-adipocytes differentiation and proliferation ↑ Adipocyte “browning” ↑ Adipocyte lipolysis	[139]
Colorectal	miR-146b-5p	↑ Adipocyte “browning” ↑ Adipocyte lipolysis ↓ Mouse weight	[107]
Lung	PTHrP	↑ HSL levels ↑ Adipocyte “browning” ↑ Glycerol release ↑ Mouse WAT lipolysis	[108]
Lung	IL-6	↑ Adipocyte lipolysis	[11]
Lung	EIF5A	↑ Adipocyte lipolysis	[109]
Lung	-	↑ Adipocyte “browning” ↑ Glycerol release ↑ Mouse WAT lipolysis	[140]
Lung/ Colon	-	↑ Adipocyte lipolysis ↓ Mouse iWAT and eWAT size	[141]
Leukemia	miR-92a-3p	↓ Adipocyte adipogenesis ↓ Mouse weight	[142]
Lung	-	↓ AMSCs adipogenesis	[143]
Prostate	-	EMT transition in AMSCs	[144]
Gastric	-	↑ AMSCs migration and invasion	[145]

↓ Decrease; ↑ increase. EV: Extracellular vesicle; Linc-ROR: long intergenic non-coding ROR; PTHrP: parathyroid hormone-related protein; HSL: hormone-sensitive lipase; WAT: white adipose tissue; IL-6: interleukin-6; EIF5A: eukaryotic translation initiation factor 5A; iWAT: inguinal WAT; eWAT: epididymal WAT; AMSC: adipose tissue-derived mesenchymal stem cells; EMT: epithelial-mesenchymal transition.

It has been shown that proteins and nucleic acids carried by EVs derived from breast^[106,133,134]^, pancreatic^[135-137]^, gastric^[110,138,139,145]^, colorectal^[107,141]^, lung^[11,108,109,140,141,143]^, and prostate^[144]^ cancers, and leukemia^[142]^ could contribute to WAT transdifferentiation and metabolism remodeling [Table 1]. For instance, in breast cancer, EVs carrying the microRNA1304-3p (miR-1304-3p) could induce the browning of white adipocytes by reducing the expression of the GATA2 transcription factor^[133]^. Moreover, the proliferation of breast cancer cells could be increased when in co-culture with the transdifferentiated adipocytes. Interestingly, the injection of miR-1304-3p positive breast cancer cells into mice led to an increased accumulation of adipocytes in the primary tumor tissue^[133]^. Similarly, the injection of miR-204-5p-positive EVs in mice with orthotopic tumors, previously induced by injecting breast cancer cells in the mammary fat pad of these animals, increased the levels of leptin and hypoxia-inducible factor 1α (HIF-1α), concomitant with increased lipolysis in their white fat depots^[106]^. Furthermore, breast cancer-derived EVs carrying miR-155 induced a decrease in the lipid droplet content and an upregulation of UCP1 protein and HSL and ATGL enzymes in white adipocytes^[134]^. Notably, in animal models, injecting these miR-155-positive EVs resulted in body weight loss and downregulation of the levels of ubiquilin 1 (UBQLN1) protein in white adipocytes, inducing its browning^[134]^. In pancreatic cancer, tumor-derived exosomes carrying the long intergenic non-coding ROR (linc-ROR) induced the downregulation of PPARγ, Glut-4, and HSL in adipocytes, resulting in the dedifferentiation of white adipocytes^[135]^. In addition, exosomes from pancreatic cancer cells, enriched in adrenomedullin hormone, could activate the p38 and the Ras-dependent extracellular signal-regulated kinase/mitogen-activated protein kinase (ERK/MAPK) signaling pathways in adipocytes. This led to an increase in HSL phosphorylation and lipolysis in white adipocytes^[136]^. Moreover, lipidomic analysis of adipocytes treated with pancreatic cancer-derived exosomes revealed a significant decrease in the triglyceride content of these adipocytes and an increase in the levels of released glycerol^[137]^. Additionally, mice injected with these exosomes exhibited smaller abdominal adipocytes, associated with elevated levels of IL-6 in circulation^[137]^. In gastric cancer, it was found that cachectic patients had high levels of miR-410-3p in their exosomes^[138]^. *In vitro* experiments showed that this miR-410-3p inhibited adipogenesis and lipid accumulation and regulated the expression of the insulin receptor substrate-1 (IRS-1), important for adipocyte differentiation. The authors hypothesized that the accentuated weight loss in these patients was correlated with the high levels of miR-410-3p^[138]^. Similarly, colorectal cancer exosomes enriched in miR-146-5p were able to regulate lipolysis and induce WAT browning*,* resulting in the weight loss of animal models^[107]^. It was also found that exosomes from patients with colorectal cancer contained high levels of this miR-146-5p^[107]^. In the same line of these results, in gastric and lung cancer models, exosomal miR-425-3p negatively impacted pre-adipocyte proliferation and differentiation, inducing a brown fat-like phenotype in fully differentiated white adipocytes through cyclic AMP/protein kinase A (cAMP/PKA) signaling pathway activation^[139]^. Similar effects were found when studying the impact of EVs released by Lewis lung carcinoma (LLC) and colon cancer cells in increasing adipocyte lipolysis. These EVs led to a decreased inguinal WAT (iWAT) and epididymal WAT (eWAT) size with increased UCP1 and PGC-1α levels in these tissues^[141]^. In addition, inhibiting the levels of the CXCR2 receptor and nuclear factor-κB (NF-κB) in adipocytes significantly reduced the lipolysis induced by IL-8-positive EVs^[141]^. Other authors have also reported that the exposure of white adipocytes to LLC-derived exosomes promoted a brown fat-like phenotype in these cells, characterized by high levels of UCP1 and HSL proteins and high glycerol secretion^[108,140]^. Furthermore, these exosomes stimulated lipolysis in the WAT depots of LLC tumor-bearing mice^[108,140]^. It was also demonstrated that LLC-derived EVs transporting the PTHrP protein could activate the PKA signaling pathway in adipocytes and consequently increase HSL levels^[108]^. These EVs also induced adipocyte lipolysis by activating the STAT3 signaling pathway through IL-6 delivery to white adipocytes^[11]^. In addition, lipolysis in adipocytes could be stimulated by the treatment with LLC-derived EVs enriched in the eukaryotic translation initiation factor 5A (EIF5A) via CREB-binding protein activation^[109]^. In brief, these reports show that tumor-derived EVs play a crucial role in reprogramming white adipocytes in different types of cancer. They can influence adipogenesis, lipolysis, and WAT browning, affecting overall adipocyte metabolism.

Interestingly, these EVs can also affect adipocytes in their earlier, more primitive form, as adipose-derived mesenchymal stem cells (AMSCs). Indeed, in lung cancer, inhibition of the transforming growth factor-beta (TGF-β) signaling pathway effectively reversed the inhibitory effects of lung cancer exosomes on AMSC adipogenesis^[143]^. Moreover, the intravenous injection of mice with gastric cancer exosomes carrying miR-155 could lead to an inhibition of AMSC adipogenesis, promoting WAT browning, which resulted in significant weight loss and the development of a cancer cachexia phenotype in these animals^[110]^. In chronic myeloid leukemia, exosomes transporting miR-92a-3p released by tumor cells were also able to suppress adipogenesis in AMSCs, by inhibiting the levels of the C/EBPα factor, which resulted in a significant weight loss in mice. In another study, prostate cancer-derived exosomes were involved in the neoplastic transformation of AMSCs, promoting the epithelial-mesenchymal transition (EMT) in recipient AMSCs by downregulating the large tumor suppressor homolog 2 (Lats2) and the programmed cell death protein 4 (PDCD4), both *in vitro* and *in vivo*^[144]^. Lastly, it was found that gastric adenocarcinoma-derived exosomes could increase the migration and invasion capacities of AMSCs by upregulating circ_0004303 RNA expression, thereby activating the activated leukocyte cell adhesion molecule (ALCAM) transmembrane protein^[145]^.

Collectively, these results emphasize the pivotal role of EVs in the interplay between tumor cells and adipocytes. Interestingly, inhibiting the production and secretion of exosomes negatively influenced the lipolysis of white adipocytes both *in vitro* and *in vivo*. Indeed, the inhibition of LLC-derived exosome biogenesis using GW4869, a pharmaceutical agent that inhibits exosome formation, suppressed the WAT browning *in vivo*^[140]^. Moreover, the knockdown of the Rab27A protein in LLC tumor-implanted mice attenuated WAT browning and lipolysis in these animals^[108]^. It was also found that the loss of adipose tissue in LLC tumor-bearing mice could be attenuated by the administration of omeprazole, an inhibitor of HSP70 and HSP90 positive EVs release by blocking Rab27b synthesis in tumor cells^[146]^. In addition, the release of EVs and IL-6 from colon cancer cells could be mitigated by treating tumor cells with atractylenolide I (an EV biogenesis inhibitor by regulating the STAT3 pathway in tumor cells), and consequently attenuate weight loss in tumor-bearing mice^[147]^. Furthermore, white adipocyte lipolysis was reduced by treating these cells with the conditioned medium from colon cancer cells pre-treated with atractylenolide I^[147]^. These observations suggest that inhibiting the biogenesis and secretion of cancer EVs could be an effective strategy against cancer cachexia.

Altogether, these findings highlight the crucial role of EVs as important mediators of tumor-AT crosstalk. Tumor-derived EVs, carrying specific miRNAs and proteins, can induce a metabolic remodeling of white adipocytes, leading to increased lipolysis and, therefore, to a higher availability of free FA and glucose in the extracellular space. These metabolites become accessible to tumor cells and can be used for energy production and membrane biosynthesis, supporting tumor growth and metastasis formation. Furthermore, these nanovesicles can promote the transdifferentiation/browning of white adipocytes by increasing mitochondrial activity, which in turn supports tumor progression. Notably, inhibitors of EV biogenesis showed to be promising in mitigating WAT browning and weight loss in animal models.

### Cancer extracellular vesicles affect adipocyte differentiation and the tumor microenvironment

Tumor cell-derived EVs also play an important role in the formation of an environment propitious to tumor progression. Tumor-derived EVs carry growth factors and enzymes that can stimulate the secretion of pro-inflammatory cytokines by adipocytes, potentially leading to tumor growth and increased angiogenesis. Furthermore, these EVs can promote tumor progression by enhancing the differentiation and motility of adipocytes in their precursor state [Table 2].

**Table 2:** Effects of cancer extracellular vesicles in adipocyte differentiation and tumor microenvironment

**Cancer type**	**Cancer EV cargo**	**Biological function**	**Ref.**
Hepatocarcinoma	-	↑ IL-6, IL-8 and MCP-1 secretion ↑ Tumor growth, angiogenesis, and macrophage recruitment *in vivo*	[148]
Breast	TGFβ	AMSCs differentiation ↑ VEGF production	[149]
Breast/ovarian	-	AMSCs differentiation ↑ TGF-β receptors in AMSCs	[150,151]
Ovarian	piR-25783	Omentum-derived fibroblasts differentiation	[152]
Liposarcoma	MDM2	↑ Proliferation, migration, and MMP2 production in pre-adipocytes	[153]

↓ Decrease; ↑ increase. EV: Extracellular vesicle; IL: interleukin; MCP-1: macrophage chemoattractant protein; AMSCs: adipose-derived mesenchymal stem cells; VEGF: vascular endothelial growth factor; TGF-β: transforming growth factor-beta; MDM2: mouse double minute 2; MMP: matrix metalloproteinase;

Indeed, exosomes released by hepatocarcinoma cells could activate the NF-κB signaling pathway in adipocytes, thereby inducing an inflammatory phenotype in these cells. The inflammatory state promoted the secretion of pro-inflammatory cytokines, including IL-6, IL-8, and macrophage chemoattractant protein (MCP-1), in a dose-dependent manner^[148]^. This pro-inflammatory microenvironment fostered tumor growth, enhanced angiogenesis, and promoted macrophage infiltration in mouse models^[148]^. Interestingly, it has also been shown that breast cancer-derived EVs induced tumor progression through AMSC differentiation into myofibroblasts, which contributed to extracellular matrix remodeling and angiogenesis^[149,150]^. AMSC differentiation was mediated through the activation of the MAPK signaling pathway by breast cancer EVs carrying the transforming growth factor-beta (TGF-β)^[149]^. Similar results were reported when investigating the impact of breast and ovarian cancer EVs in AMSC differentiation^[150,151]^. Increased levels of the TGF-β receptor were found in AMSCs treated with breast and ovarian cancer-derived EVs. These findings indicate that these particles could activate TGF-β receptor-mediated signaling pathways, leading to the differentiation of AMSC into myofibroblast, creating a favorable microenvironment for tumor cell growth^[150,151]^. The transfer of piR-25783 from ovarian cancer exosomes to omentum-derived fibroblasts also promoted the differentiation of the fibroblasts to myofibroblasts and consequently contributed to the formation of a microenvironment propitious to the establishment and growth of tumor cells in the omentum^[152]^. Furthermore, it was observed a decrease in p53 tumor suppressor activity and an increase in proliferation, migration, and production of matrix metalloproteinase 2 (MMP2) of pre-adipocytes exposed to EVs secreted from liposarcoma, carrying mouse double minute 2 (MDM2) DNA, thereby contributing to the establishment of a pre-metastatic niche that facilitates tumor growth and colonization^[153]^.

These studies suggest that EVs play a crucial role in establishing a favorable microenvironment for tumor growth by inducing an inflammatory state and promoting the differentiation of AMSCs into myofibroblasts, remodeling the extracellular matrix, and stimulating angiogenesis. These mechanisms could contribute to the formation of a pre-metastatic niche that facilitates tumor progression and metastasis formation. The mentioned studies are not directly correlated with cancer cachexia. Nevertheless, it is important to note that tumor metastasis is usually associated with advanced stages of cancer and closely linked to the development of cancer cachexia.

### Adipocyte-derived extracellular vesicles promote tumor progression and metabolic reprogramming

After being transdifferentiated by cancer cells, adipocytes secrete factors, including metabolites, adipokines, and EVs (transporting FAO-related proteins and enzymes, miRNAs, and lncRNAs) that can affect the tumor microenvironment and the biology of tumor cells. The role of adipocyte-derived EVs in AT-cancer crosstalk [Table 3] has been observed in different types of cancer with an impact on tumor progression, metastasis formation, and treatment resistance^[167-169]^. These observations have been reported in EVs isolated from naïve adipocytes and by EVs isolated from adipocytes in obese or diabetic conditions, highlighting the impact of specific metabolic disorders in cancer progression.

**Table 3:** Effects of adipocyte-derived extracellular vesicles in tumor progression and metabolic reprogramming

**Cancer type**	**Adipocyte EV cargo**	**Biological impact**	**Ref.**
Melanoma	FAO‐related proteins	↑ FAO on tumor cells ↑ Tumor cell invasion and migration Mitochondria reorganization	[116,117]
Melanoma	β-catenin	↑ Tumor cell progression and aggressiveness	[112]
Breast	TSP5	↑ EMT induction Changes in tumor cell morphology	[154]
Breast	-	↑ Tumor cell invasion and migration ↑ EMT induction	[114]
Breast	-	↑ Tumor cell migration	[155]
Breast	Leptin and MMP9	↑ MMP9 secretion ↑ Tumor cell proliferation and motility	[156]
Breast	-	↓ Tumor cell apoptosis ↑ Tumor cell proliferation and migration	[157]
Breast	miR-155-5p, miR-10a-3p, and miR-30a-3p	↑ Tumor cell proliferation ↑ Mitochondrial density	[113]
Nasopharyngeal	↓ miR-433-3p	↑ Tumor cell motility ↑ Lipid accumulation	[158]
Lung	MMP3	↑ Tumor cell invasion and metastasis formation *in vivo*	[159]
Liver	-	↑ Production of liver fibrosis genes, integrins, and MMP9	[160]
Hepatocellular	miR-32a/b	↑ Tumor cell proliferation and migration 5-FluoroUracile resistance	[118]
Ovarian	miR-21	↓ Tumor cell apoptosis Paclitaxel resistance	[119]
Ovarian	-	↑ Tumor cell growth ↑ Metastasis formation *in vivo*	[161]
Colorectal	MTTP	↓ Tumor cell ferroptosis Oxaliplatin resistance	[115]
Colorectal	-	↑ Tumor cell invasion and migration	[162]
Prostate	-	↑ Tumor cell proliferation and migration Docetaxel resistance Tumor cell metabolism switch	[120]
Prostate	-	EMT transition	[163]
Myeloma	LncRNAs (LOC606724 and SNHG1)	Bortezomib resistance	[164]
Pancreatic	-	↑ Tumor cell proliferation and motility ↑ EMT induction	[135,165]
Osteosarcoma	-	↑ Tumor cell proliferation, migration, and invasion	[166]

↓ Decrease; ↑ increase. EV: Extracellular vesicle; FAO: fatty acid β-oxidation; TSP5: thrombospondin-5; EMT: epithelial-mesenchymal transition; MMP: matrix metalloproteinase; MTTP: microsomal triglyceride transfer protein; lncRNAs: long non-coding RNAs.

Recent studies have shown that adipocyte-derived EVs can reprogram the metabolism of melanoma cells toward FAO by providing FA, proteins, and the necessary enzymes for this metabolic transformation^[116,117]^. Moreover, these EVs could promote the migration and invasion capacity of melanoma cells^[116,117]^ by stimulating the reorganization of mitochondria in these tumor cells^[116]^. Interestingly, it was observed that adipocytes from obese mice released higher amounts of EVs compared to those from lean mice^[116,117]^. This higher secretion of adipocyte-derived EVs in obese mice may lead to a stronger effect on tumorigenesis since the amount of FA delivered to tumor cells increases. Another study revealed that EVs derived from the AT of obese patients could regulate the expression of the TWIST1 transcription factor in prostate cancer cells and thus regulate the EMT transition of these cells^[163]^. Moreover, it was also shown that adipocyte EVs carrying β-catenin could block the transcription of CDKN2A factor (responsible for cell growth regulation) and decrease p16^INK4A^ levels (associated with cell cycle regulation) in melanoma cells, which resulted in tumor progression^[112]^. The authors hypothesize that the higher secretion of EVs containing β-catenin in obese patients might amplify the effect of these particles on tumor aggressiveness^[112]^. Furthermore, EVs isolated from the adipocytes of obese patients, enriched in leptin and MMP9, could promote the release of MMP9 by breast cancer cells^[156]^. This could enhance the proliferation and mobility of tumor cells by activating the ERK/MAPK and the PI3K/AKT signaling pathways^[156]^. Remarkably, EVs isolated from obese individuals promoted the proliferation of breast cancer cells by enhancing the mitochondrial function and density of these cells, through the Akt/mTOR/P70S6K signaling pathway^[113]^. Interestingly, it was found that the enriched miRNAs (miR-155-5p, miR-10a-3p, and miR-30a-3p) in these EVs stimulated oxidative phosphorylation (OXPHOS) in tumor cells^[113]^. In liver cancer, the TGF-β signaling pathway was dysregulated in hepatocytes by the presence of adipocyte EVs isolated from obese patients^[160]^. The treatment of hepatocytes with these EVs led to an increased expression of genes involved in the development of liver fibrosis, including the tissue inhibitor of matrix metalloproteinase-1 and -4 (TIMP-1 and -4), the integrins ανβ-5 and ανβ-8, and MMP9, thus evidencing the role of adipocyte EVs in modulating the tumor microenvironment^[160]^. Similarly, EVs carrying thrombospondin-5 (TSP5) protein, isolated from patients with type 2 diabetes, could increase the expression of genes associated with EMT transition in breast cancer cells, leading to alterations in cellular morphology^[154]^. Furthermore, it was found that adipocyte-derived EVs could enhance the growth, motility, and invasion of breast cancer cells by stimulating the activity of HIF-1α in tumor cells^[114]^. Additionally, it was observed that these EVs could facilitate the process of lung metastatic colonization in mice after breast cancer cells were injected into the tail vein of these animals^[114]^. These studies illustrate the link between obesity, diabetes, and cancer progression. Adipocyte-derived EVs from obese or diabetic individuals could contribute to microenvironment modulation, tumor aggressiveness, and metastasis. Indeed, it has been described that obesity is strongly linked to a higher risk of developing several types of cancer (as reviewed in ref^[170]^). In obesity, AT produces elevated levels of estrogen, pro-inflammatory cytokines (TNFα, IL-6, IL-1β), adipokines (leptin), and EVs^[171]^, which may contribute to enhanced tumor invasiveness, proliferation, and metastasis^[172]^. In addition, obesity has been associated with an increased risk of overall mortality and disease recurrence in patients with breast^[173]^, liver^[174]^, colorectal^[175,176]^, prostate^[177]^, and pancreatic^[178]^ cancer. Both obesity and cancer cachexia share common underlying mechanisms that result in profound metabolic alterations. Key factors such as insulin resistance^[179]^, WAT lipolysis^[180]^, muscle atrophy^[181]^, and systemic inflammation^[182]^ play crucial roles in both conditions. Interestingly, a recent study demonstrated that obese and lean LLC-bearing mice experience similar patterns of weight loss^[183]^. Moreover, obese mice exhibit reduced survival rates and mitochondrial dysfunction, emphasizing the complex interplay between obesity and cancer outcomes^[183]^. Obesity not only increases the risk of cancer mortality and recurrence but also significantly increases the risk of patients developing type 2 diabetes (as reviewed in ref^[184]^). In diabetic patients, insulin resistance, elevated levels of glucose, insulin, and IGF-1, as well as pro-inflammatory cytokines, increased leptin, and decreased adiponectin can increase the risk of cancer development^[185]^. Evidence has shown that diabetes type 2 is linked to a higher incidence and recurrence rates of pancreatic^[186,187]^, liver^[186,187]^, colorectal^[187,188]^, breast^[187,189]^, and endometrial^[186]^ cancer. Importantly, it was found that the pre-existence of type 2 diabetes in patients with pancreatic and colorectal cancer could lead to increased cachexia incidence, resulting in higher weight loss and reduced survival rates^[190]^. These findings show the complex metabolic interplay between obesity, type 2 diabetes, and cancer cachexia, emphasizing the need for novel and better comprehensive management strategies to address the metabolic unbalance in these patients.

Furthermore, breast cancer cell migration could also be enhanced by AMSCs-derived exosomes through the activation of the tumor Wnt/β-catenin signaling pathway^[155]^. It was also noted that exosomes secreted from AMSCs reduced apoptosis and promoted the proliferation and migration of breast cancer cells through activation of the Hippo signaling pathway^[157]^. In the same line with these results, adipocyte-derived exosomes expressing low levels of miR-433-3p promoted lipid accumulation in nasopharyngeal cancer cells by activating the stearoyl-CoA desaturase 1 (SCD1), a key enzyme in FA metabolism^[158]^. It was also found that adipocyte exosomes enriched in MMP3 could activate MMP9 in lung tumor cells and promote their invasiveness and metastasis *in vivo*^[159]^. Studies have also shown that adipocytes could regulate insulin resistance in hepatocytes by secreting EVs carrying miR-141-3p^[191]^, monocyte chemoattractant protein-1 (MCP-1), IL-6, and macrophage migration inhibitory factor (MIF)^[192]^. The weak response of cells to insulin translated into increased levels of extracellular glucose that could be taken up by tumor cells and used as an energy source^[193]^. Moreover, exosomes from AMSCs were able to promote ovarian cancer cell growth and motility *in vitro* and metastasis formation in mouse models by regulating the levels of the forkhead box protein M1 (FOXM1) protein^[161]^. The increased presence of this protein could induce the expression of Cyclin F and kinesin family member 20A (KIF20A) proteins as well as the activation of the ERK1/2 and c-Jun N-terminal kinases (JNK)-MAPK signaling pathways in tumor cells^[161]^. Furthermore, exosomes from colon cancer cells could induce the differentiation of AMSCs into cancer-associated fibroblasts (CAFs) by activating the NF-κB signaling pathway through the transient receptor potential cation channel subfamily C member 3 (TRPC3)^[162]^. These activated CAFs could further promote the migration and invasion of colon cancer cells. Moreover, high levels of TRPC3 in mesenchymal cells were associated with a worse clinical outcome in colon cancer patients^[162]^. Additionally, pancreatic tumor cells could induce morphological and metabolic alterations in adipocytes, which in turn led to increased motility and proliferation of tumor cells^[135,165]^. The treatment of osteosarcoma cells with AMSC-derived exosomes could induce their proliferation, migration, and invasion by upregulating the collagen beta(1-O)galactosyltransferase 2 levels in tumor cells, a gene responsible for the collagen glycosylation in the endoplasmic reticulum^[166]^. In the case of ovarian cancer, adipocytes could promote a metabolic switch of tumor cells favoring β-oxidation by direct transfer of lipids, which contributed to tumor growth^[168]^. Similar results were reported when EVs secreted by white adipocytes could enhance the migration, invasion, and proliferation of prostate cancer cells^[120]^. Interestingly, after exposing prostate cancer cells to adipocyte-derived EVs, the authors observed a switch in tumor cell metabolism characterized by increased glucose consumption and lactate and ATP production^[120]^. Briefly, this evidence shows that adipocyte-derived EVs have a significant impact on cancer progression by modulating the metabolism of tumor cells and enhancing their malignancy.

It has also been shown that adipocyte-derived exosomes can contribute to therapy resistance in different types of cancer. For instance, adipocyte-derived exosomes containing miR-32a and miR-32b could activate the von Hippel-Lindau-HIF-1α (VHL-HIF-1α) pathway in hepatocellular cancer cells, leading to increased resistance to 5-FluoroUracile^[118]^. Interestingly, high levels of these exosomes were found in hepatocellular carcinoma patients, and a low survival rate was associated with low VHL presence and high HIF-1α^[118]^. In another study, the downregulation of apoptotic protease activating factor 1 (APAF1) by adipocyte-derived exosomes carrying miR-21 suppressed ovarian cancer cell apoptosis and conferred resistance to paclitaxel treatment^[119]^. In colorectal cancer, ferroptosis of tumor cells could be inhibited by adipocyte-derived exosomes carrying the microsomal triglyceride transfer protein (MTTP)^[115]^. Additionally, the exposure of colorectal cancer cells to adipocyte-derived exosomes was able to induce the resistance of tumor cells to oxaliplatin treatment^[115]^. Moreover, increased levels of adipocyte exosomal long non-coding RNAs (lncRNAs) LOC606724 and SNHG1 were associated with a poor prognosis in multiple myeloma patients^[164]^. It was also observed that these lncRNAs induced bortezomib resistance in tumor cells^[164]^. Similarly, EVs released by white adipocytes could negatively impact the sensitivity of prostate cancer cells to docetaxel treatments^[120]^.

Altogether, these findings suggest that adipocyte-derived EVs are key players in the metabolic and phenotypic reprogramming of tumor cells, namely by affecting tumor biology, drug resistance, cancer progression, and metastasis formation. Importantly, metastatic tumors can contribute to systemic inflammation and metabolic dysfunctions, exacerbating cancer cachexia^[194]^. Furthermore, it has been reported that obesity may lead to an increased secretion of adipocyte-derived EVs, thereby enhancing the effects of these nanoparticles in tumor progression.

### Adipose tissue-derived extracellular vesicles as inhibitors of cancer progression

So far, we discussed the role of adipocyte-derived EVs in remodeling the metabolism and acquired aggressiveness of tumor cells. However, it is worth mentioning that some studies have also reported that these EVs could inhibit the growth and progression of tumor cells by transporting and delivering specific miRNAs into the recipient cell [Table 4].

**Table 4:** Adipocyte-derived extracellular vesicles as inhibitors of cancer progression

**Cancer type**	**Adipocytes EV cargo**	**Biological function**	**Ref.**
Prostate	miR-145	↓ Tumor cell proliferation and invasion ↓ Metastasis formation *in vivo*	[195]
Ovarian	Anticancer miRNAs	↓ Tumor cell proliferation ↑ Tumor cell apoptosis	[196]
Lung	miR-99a-5p	↓ Tumor cell proliferation and migration	[197]
Neuroblastoma	LncRNAs LINC00622	↓ Tumor cell proliferation, migration, and invasion	[198]
Glioblastoma	-	↓ Tumor cell proliferation ↓ Tumor invasion *in vivo*	[199]
Colorectal	-	↓ EGFR and aquaporin 5 levels	[200]

↓ Decrease; ↑ increase. EV: Extracellular vesicle; lncRNAs: long non-coding RNAs; EGFR: epidermal growth factor receptor.

In prostate cancer, AMSC cells carrying the tumor suppressor miR-145 could inhibit the growth and invasion of tumor cells *in vitro* through direct contact or mediated by EVs^[195]^. In addition, the metastasis of these cells could be mitigated by treating mouse models with miR-145-positive AMSCs^[195]^. In lung cancer, adipocyte-derived exosomes enriched in miR-99a-5p from obese mice could mitigate the proliferation and migration of tumor cells^[197]^. In another cell model, AMSCs-derived exosomes carrying the lncRNA LINC00622 inhibited the proliferation, migration, and invasion of tumor cells by regulating the expression of the transcriptional factor androgen receptor and consequently promoted the activation of the gamma-aminobutyric acid type B receptor subunit 1 (GABBR1)^[198]^. In glioblastoma, it was also observed that the treatment of tumor cells with EVs derived from AMSCs led to a decrease in the proliferation capacity of these cells *in vitro* and negatively impacted their invasion capacity *in vivo*^[199]^. In colorectal cancer, it was found that AMSCs-derived exosomes could significantly reduce the mRNA levels of epidermal growth factor receptor (EGFR) and aquaporin 5, which plays an important role in cancer progression by promoting tumor cell motility^[200]^. Thus, the authors defend that a good strategy against tumor progression may involve inhibiting the levels of this transmembrane protein using these AMSCs-derived exosomes^[200]^. In ovarian cancer, it was also reported the impact of AMSCs-derived adipocytes on the inhibition of tumor cell proliferation and induction of apoptosis by promoting the activation of the p53, Bcl-2-associated X protein (BAX), and caspase 3 and 9 via anticancer miRNAs^[196]^.

Overall, these studies demonstrate the potential of EVs derived from AMSCs as therapeutic strategies for inhibiting the growth and progression of various types of cancer. By carrying specific miRNAs and lncRNAs, these EVs could inhibit the proliferation, migration, and invasion of tumor cells. These findings suggest that AMSCs-derived EVs could be a valuable tool for the development of new anticancer therapies.

### Adipose tissue-derived extracellular vesicles as potential therapeutic delivery strategies

As previously mentioned, AMSCs-derived EVs hold great promise as a novel anticancer strategy. Recent evidence demonstrates the use of targeted manipulation of these nanoparticles to inhibit tumor progression and enhance chemosensitivity in tumor cells. After being loaded with specific miRNAs, EVs showed the potential to trigger tumor cell apoptosis, decrease tumor growth, and metastasis formation, and enhance the sensitivity of tumor cells to anticancer treatments [Table 5].

**Table 5:** Adipocyte-derived extracellular vesicles as potential therapeutic delivery strategies

**Cancer type**	**Manipulated EV cargo**	**Biological function**	**Ref.**
Breast	miR-145	↓ Metastasis formation *in vivo*	[201]
Breast	↓ CD90 and miR-16-5p	↓Tumor cell growth ↑ Tumor cell apoptosis ↓ Tumor mass *in vivo*	[202]
Breast	miR-424-5p	↑ Anti-inflammatory cytokines secretion ↓ Tumor cell growth ↑ Tumor cell apoptosis	[203]
Breast	miR-381-3p	↓ Tumor cell proliferation, migration, and invasion ↑ Tumor cell apoptosis	[204]
Breast	miR-140	↓ Tumor cell migration, stemness, and differentiation ↓Tumor growth *in vivo*	[205]
Breast	miR-1236	↑ Cisplatin sensitivity	[206]
Hepatocellular	miR-199a	↑ Doxorubicin sensitivity ↓ Tumor cell viability	[207]
Hepatocellular	miR-122	↑ 5-FluoroUracile and sorafenib sensitivity	[208]
Blader	miR-138-5p	↓ Tumor cell proliferation, migration, and invasion ↓ Tumor growth *in vivo*	[209]

↓ Decrease; ↑ increase. EV: Extracellular vesicle.

In breast cancer, lentivirus transfection was used to load miR-145 into AMSCs^[201]^. These manipulated AMSCs were able to transfer miR-145 via EVs into breast cancer cells, which negatively impacted the expression of genes related to cell apoptosis and metastasis in tumor cells. These included *MMP9*, Erb-B2 receptor tyrosine kinase 2 (*ERBB2*), tumor suppressor *p53*, and Rho-associated coiled-coil containing protein kinase 1 (*ROCK1*)^[201]^. Furthermore, the injection of AMSCs-derived EVs containing low levels of CD90 in mice bearing breast cancer cell xenografts, slower tumor growth and reduced tumor mass formation in these animals^[202]^. In addition, loading the miR-16-5p into AMSC EVs via liposomes enhanced the effect of these CD90 low-EVs in inducing tumor cell apoptosis *in vitro* and reduced tumor mass *in vivo*^[202]^. Similar results were observed in a triple-negative breast cancer cell model. AMSCs were transfected with miR-424-5p using lipofectamine reagents^[203]^. The miR-424-5p transfer via EVs led to the suppression of programmed death-ligand 1 (PD-L1) levels, consequently slowing tumor growth and promoting apoptosis of breast cancer cells. Additionally, miR-424-5p transfer via EVs induced the secretion of anti-inflammatory cytokines and diminished the release of pro-inflammatory cytokines^[203]^. Moreover, the treatment of triple-negative breast cancer cells with AMSCs-derived exosomes loaded with miR-381-3p, by electroporation, inhibited the proliferation, migration, and invasion of the breast cancer cells and promoted their apoptosis^[204]^. It was found that these exosomes could decrease the levels of the Wnt pathway and EMT-related genes (Snail, CTNNB1, and N-cadherin)^[204]^. In bladder cancer, exosomes derived from AMSCs previously transfected with a lentivirus carrying the miR-138-5p gene, have shown promising results in reducing tumor cell proliferation, migration, and invasion^[209]^. Notably, the growth and spread of tumor cells both *in vitro* and *in vivo* were reduced by AMSCs-derived exosomes carrying the miR-138-5p^[209]^. Interestingly, the invasion capacity and viability of skin cancer cells were reduced upon treating these cells with AMSCs-derived exosomes carrying the miR-199a-5p^[210]^. It was found that these miR-199-5p-positive EVs control the expression of the SOX4 transcription factor^[210]^. It was further suggested that exosomes secreted from pre-adipocytes could regulate early-stage breast cancer cells. The treatment of white pre-adipocytes with anti-tumor component shikonin (SK), led to the secretion of exosomes with high levels of miR-140. These exosomes further inhibited the migration, stemness, and differentiation of tumor cells by decreasing the levels of SOX9 protein^[205]^, an important transcription factor of pre-adipocyte differentiation^[211]^. Notably, the injection of SK-treated adipocyte exosomes reduced breast tumor volume compared to mice injected with untreated exosomes^[205]^. This finding suggests that the manipulation of EVs derived from AMSCs has therapeutic potential against different cancer types, effectively suppressing tumor growth and metastasis formation. In particular, the incorporation of specific miRNAs into these EVs seems to enhance their anti-tumor effects.

It was also proposed that these manipulated EVs could play a significant role in enhancing the sensitivity of tumor cells to chemotherapeutic drugs. Indeed, the sensitivity of breast cancer cells to cisplatin could be increased by treating these cells with AMSC-derived exosomes enriched in miR-1236^[206]^. This miR-1236 regulated the levels of SLC9A1 in breast cancer cells, thereby affecting the Wnt/β-catenin pathway^[206]^. Furthermore, AMSCs-derived exosomes carrying miR-199a^[207]^ and miR-122^[208]^ increased the sensitivity of hepatocellular cancer cells to chemotherapeutic agents (doxorubicin, 5-FluoroUracile, and sorafenib) *in vitro* and *in vivo*. The transfer of miR-199a via exosomes inhibited the mTOR signaling pathway, leading to a decrease in tumor cell viability. AMSCs were transfected with a lentivirus or a plasmid containing pre-miR-199a-3p^[207]^ or miR-122^[208]^, respectively.

Currently, a clinical trial is ongoing where manipulated EVs derived from mesenchymal stromal cells are being used to treat patients with pancreatic cancer^[212]^. In this phase I clinical trial, metastatic pancreatic ductal adenocarcinoma patients with the KRAS^G12D^ mutation were treated with mesenchymal stromal cell-derived exosomes loaded with KRAS^G12D^ siRNA. Initial results from the trial indicate that the treatment is being well-tolerated at lower doses, leading to reduced levels of circulating Kras^G12D^ DNA and phosphorylated ERK in the tumor^[213]^.

In summary, these findings highlight the potential of adipocyte-derived EVs, carrying specific miRNAs, as innovative therapeutic strategies against cancer. The engineered EVs have exhibited success in inducing tumor cell apoptosis, slowing tumor progression and metastasis formation, and enhancing the response to therapy in animal models. Further research is essential to fully understand the potential of EV-based therapies in clinical settings.

## CONCLUSION

Cancer cachexia is a complex syndrome involving skeletal muscle and body fat loss, that has been intricately linked to elevated morbidity and mortality rates^[3]^. Despite nutritional interventions, a complete reversal of cachexia remains elusive, and effective therapies for cancer cachexia patients are currently lacking^[3]^. Consequently, unraveling the intricate molecular mechanisms underlying the development and progression of cancer cachexia will potentiate the development of innovative therapeutic approaches. This review explores the latest insights into the role of EVs in modulating the dynamic interplay between tumor cells and adipose tissue in the context of cancer cachexia.

Recent studies have highlighted that tumor cells release EVs capable of influencing adipocyte recipient cell behavior, thus establishing a crucial link in the tumor-AT crosstalk. Tumor-derived EVs can induce a brown fat-like phenotype by stimulating adipocyte lipolysis and the secretion of metabolites, such as FA. Additionally, these adipocytes reciprocate by releasing EVs carrying lipids, proteins, and miRNAs that can trigger metabolic reprogramming of tumor cells, favoring fatty acid β-oxidation.

Compelling evidence suggests that manipulating EVs could serve as a therapeutic strategy for cancer cachexia. In animal models, inhibiting the production and secretion of tumor-derived EVs^[108,140,147]^ or modifying the cargo carried by adipocyte-derived EVs^[207-209]^ effectively mitigated cachexia effects. In addition, these alterations were also capable of inhibiting tumor progression and enhancing tumor cells’ sensitivity to therapy. Given their capacity to transport vital information between cells and their presence in various biological fluids^[121]^, EVs emerge as a promising drug delivery system. Therefore, comprehending the pathways that regulate EV biogenesis, secretion, and cargo selection and packaging holds significant promise as a therapeutic strategy against cachexia.

In conclusion, EVs are pivotal players in the intricate communication network between tumor cells and adipocytes during cancer cachexia. However, numerous questions persist regarding the role of EVs as modulators of cancer cachexia. Future research should focus on disclosing the mechanisms and EV cargo that facilitate tumor-AT communication and modulation. It is worth noting that the current research on EV-mediated communication between tumor cells and adipose tissue relies mostly on *in vitro* observations. Therefore, there is a need for further studies using animal models and clinical samples to validate the *in vitro* findings and effectively translate the knowledge into the clinical setting. We believe that this knowledge will be instrumental in the development of novel targeted therapeutic strategies for cancer cachexia management, ultimately enhancing the quality of life and survival of cancer patients.

## DECLARATIONS

### Copyright

© The Author(s) 2024.
